# Conventional 3T brain MRI and diffusion tensor imaging in the diagnostic workup of early stage parkinsonism

**DOI:** 10.1007/s00234-015-1515-7

**Published:** 2015-04-07

**Authors:** Frederick J. A. Meijer, Anouke van Rumund, Anil M. Tuladhar, Marjolein B. Aerts, Imke Titulaer, Rianne A. J. Esselink, Bastiaan R. Bloem, Marcel M. Verbeek, Bozena Goraj

**Affiliations:** 1 Department of Radiology and Nuclear Medicine, Radboud University Nijmegen Medical Center, Geert Grooteplein 10, Postbus 9101, 6500 HB Nijmegen, The Netherlands; 2 Department of Neurology, Donders Institute for Brain, Cognition and Behaviour, Radboud University Nijmegen Medical Center, Nijmegen, The Netherlands; 3 Department of Laboratory Medicine, Radboud University Nijmegen Medical Center, Nijmegen, The Netherlands; 4 Department of Diagnostic Imaging, Medical Center of Postgraduate Education, Warsaw, Poland

**Keywords:** Brain, MRI, DTI, Parkinson’s disease, Atypical parkinsonism

## Abstract

**Introduction:**

The aim of this study is to evaluate whether the diagnostic accuracy of 3 T brain MRI is improved by region of interest (ROI) measures of diffusion tensor imaging (DTI), to differentiate between neurodegenerative atypical parkinsonism (AP) and Parkinson’s disease (PD) in early stage parkinsonism.

**Methods:**

We performed a prospective observational cohort study of 60 patients presenting with early stage parkinsonism and initial uncertain diagnosis. At baseline, patients underwent a 3 T brain MRI including DTI. After clinical follow-up (mean 28.3 months), diagnoses could be made in 49 patients (30 PD and 19 AP). Conventional brain MRI was evaluated for regions of atrophy and signal intensity changes. Tract-based spatial statistics and ROI analyses of DTI were performed to analyze group differences in mean diffusivity (MD) and fractional anisotropy (FA), and diagnostic thresholds were determined. Diagnostic accuracy of conventional brain MRI and DTI was assessed with the receiver operating characteristic (ROC).

**Results:**

Significantly higher MD of the centrum semiovale, body corpus callosum, putamen, external capsule, midbrain, superior cerebellum, and superior cerebellar peduncles was found in AP. Significantly increased MD of the putamen was found in multiple system atrophy–parkinsonian form (MSA-P) and increased MD in the midbrain and superior cerebellar peduncles in progressive supranuclear palsy (PSP). The diagnostic accuracy of brain MRI to identify AP as a group was not improved by ROI measures of MD, though the diagnostic accuracy to identify MSA-P was slightly increased (AUC 0.82 to 0.85).

**Conclusion:**

The diagnostic accuracy of brain MRI to identify AP as a group was not improved by the current analysis approach to DTI, though DTI measures could be of added value to identify AP subgroups.

## Introduction

Brain MRI is commonly performed in the diagnostic workup of parkinsonism. The main purpose of this study is to assess cerebrovascular damage for the diagnosis of vascular parkinsonism and to exclude other possible but more rare causes of parkinsonism (e.g., multiple sclerosis). It can also show abnormalities which are suggestive of neurodegenerative atypical parkinsonism (AP) [1–3]. Examples include atrophy and T2 hypo-intensity of the putamen, which can be seen in the parkinsonian form of multiple system atrophy (MSA-P), while signal intensity changes of the pons (“hot cross bun” sign) or pontocerebellar atrophy can point to the cerebellar form of MSA (MSA-C). Atrophy of the midbrain (“hummingbird” sign) or signal intensity changes in the superior cerebellar peduncles are suggestive of progressive supranuclear palsy (PSP). Asymmetrical cortical atrophy is the hallmark of Corticobasal degeneration (CBD). Conventional brain MRI is usually normal or will show age-related changes in early stage Parkinson’s disease (PD), which is the most frequent cause of parkinsonism [4]. Later on, cortical atrophy of the frontal or temporal lobe can be seen in PD.

Although certainty about the diagnosis increases during clinical follow-up, the aim of ancillary investigations is to increase certainty about the diagnosis in early disease stages, which is important for adequate patient counseling and to some extent also treatment [5]. It has been shown that the added value of conventional brain MRI in the diagnostic workup of parkinsonism is highest in case there is uncertainty about the diagnosis [6].

In recent years, new MRI techniques have become available for clinical practice, including diffusion-weighted imaging (DWI) and diffusion tensor imaging (DTI). Diffusion MRI quantifies the random movement of water molecules and seems to represent a quantitative measure of microstructural changes in neurodegenerative pathology, even when no abnormalities are seen on conventional MRI sequences. While fractional anisotropy (FA) estimates the degree of anisotropy, i.e., restriction of the random motion of water molecules by the normal architecture of glial tissue and fiber tracts, mean diffusivity (MD) is an averaged measure of diffusivity. Loss of microstructural integrity of brain tissue is commonly reflected by a decrease in FA and an increase in MD. Two main quantitative analyses for diffusion MRI include the region of interest (ROI) method and the automated voxel-based methods. Tract-based spatial statistics (TBSS) is an example of the automated voxel-based method. These two approaches yield complementary results, but each method has its drawbacks and does not completely reflect ongoing changes [7].

Different patterns of microstructural changes can be identified by DTI in PD and the different forms of AP, which seem to correlate with known histopathologic changes in these diseases [8–10]. Examples include increase in MD and decrease in FA of the putamen or pontocerebellar structures in MSA, and diffusional changes of the midbrain and superior cerebellar peduncles in PSP [10]. Previous studies indicate that DTI measures of the basal ganglia, brainstem, and cerebellum can accurately identify subjects diagnosed with PD and different forms of AP [8, 11]. Despite of the positive study results, actual application of quantitative DTI in clinical practice is limited because validated diagnostic criteria are generally lacking and no clear guidelines are available how to interpret quantitative diffusional data of the individual patient. Also, few studies evaluated brain MRI and DTI in early disease stages where the added value of brain MRI is most clinically relevant [12, 13].

Our study objective was to evaluate whether ROI measures of DTI improve the diagnostic accuracy of conventional 3 T brain MRI in the diagnostic workup of early stage parkinsonism, to differentiate between Parkinson’s disease and neurodegenerative atypical parkinsonism.

## Material and methods

### Study group

We performed a prospective observational cohort study of 60 patients presenting with parkinsonism. Patients were consecutively recruited at our outpatient movement disorder clinic in the period 2010–2012. Study inclusion criteria were clinical signs and symptoms of parkinsonism (hypokinetic-rigid syndrome of neurodegenerative origin), with an uncertain clinical diagnosis and disease duration less than 3 years. Exclusion criteria were age below 18, prior brain surgery, presence of other neurological diseases, and instable comorbidity. The medical ethics committee of our hospital approved the study and all participants gave written informed consent.

### Study design

Clinical examination of all patients was performed at baseline by an experienced physician (MA, AR) and included standardized history taking and neurological examination. Cardiovascular risk factors, activities in daily living, medication use (including response to anti-parkinsonian medication), disease onset, clinical signs, most affected body site, balance, and fear of falling were assessed. Clinical neurological scores were applied, including the Non-Motor Symptom Scale (NMSS) [14], Unified Parkinson’s Disease Rating Scale (UPDRS-III) for evaluating severity of motor symptoms [15], the Mini-Mental State Examination (MMSE) to evaluate global cognitive status [16], and Hoehn and Yahr staging scale (H&Y) for assessing disease severity [17].

At baseline, all patients had a brain MRI. After clinical follow-up, final diagnoses could be made by two experienced clinicians (AR, RE) according to international diagnostic criteria [18–24] based on neurological signs that developed during the course of the disease (as identified during repeat neurological exams), rate of disease progression, and treatment response. Using these “silver standard” diagnoses, the ability of brain MRI and DTI to differentiate between PD and AP was evaluated.

### Brain MRI scanning protocol

All patients had a 3 T brain MRI study (Magnetom Trio, Siemens, Erlangen, Germany). Total acquisition time was 42 min and included a 7-min, 24-s DTI acquisition. A 12-channel receive-only phased-array head coil was used. Conventional brain MRI included the following: 3D T1 MP RAGE (TR/TE = 2300/4.71 ms, flip angle = 12°, voxel size 1 × 1 × 1 mm, FOV = 256 mm), T2 TSE (TR/TE = 5830/120 ms, flip angle = 120°, voxel size 0.6 × 0.6 × 3 mm, FOV = 240 mm), T2 FLAIR (TR/TE = 9000/86 ms, flip angle = 150°, voxel size 0.7 × 0.6 × 5 mm, FOV = 240 mm), proton density (TR/TE = 2000/20 ms, flip angle = 90°, voxel size 0.9 × 0.9 × 3 mm, FOV = 240 mm), and DWI (TR/TE = 3900/89 ms, *b* values 0 and 1000 s/mm^2^, flip angle = 90°, voxel size 1.3 × 1.3 × 5 mm, FOV = 240 mm) sequences.

Details of the DTI sequence were as follows: single-shot spin-echo EPI, *b* values 0 and 1000 s/mm^2^, TR/TE = 13,000/102 ms, number of encoding directions = 30, FOV = 240 mm, and voxel size 2 × 2 × 2 mm.

### Imaging analysis

Two neuroradiologist (FJAM, 5 years of experience, and BG, 30 years of experience) evaluated conventional brain MRI studies in a standardized manner, blinded to clinical information. The following abnormalities were scored for the evaluation of parkinsonism [2, 3, 6, 25]: atrophy and T2 hypo-intensity of the putamen, putaminal rim sign, pontine atrophy, hot cross bun sign, cerebellar atrophy, T2 hyper-intensity, and atrophy of the middle cerebellar peduncle (MCP) were scored as indicators of MSA. Midbrain atrophy, hummingbird sign, and reduced AP midbrain diameter <14 mm were scored as indicators of PSP. Cortical atrophy and third and lateral ventricle dilatation were scored as indicators for either CBD or LBD. Furthermore, white matter T2 hyper-intensity changes and the presence of infarction were scored.

The in-house developed algorithm named “PATCH” [26] was employed to the raw DTI data to detect and correct head and cardiac motion artifacts and eddy currents using an iteratively reweighted least squares algorithm. Corrections of eddy current and motion artifacts were performed simultaneously.

First, a TBSS analysis was performed. FA and MD were calculated using DTIFit within the FSL toolbox (Functional MR Imaging of the Brain Software Library, University of Oxford, United UK; http://www.fmrib.ox.ac.uk/fsl/), which were fed into the TBSS pipeline. This pipeline includes the thinning procedure using the mean FA image to create a common skeleton, which represents the core structure of the white matter tract. The FA threshold value of 0.2 was applied to include major white matter tracts. These projection vectors were then applied to MD.

**Fig. 1:**
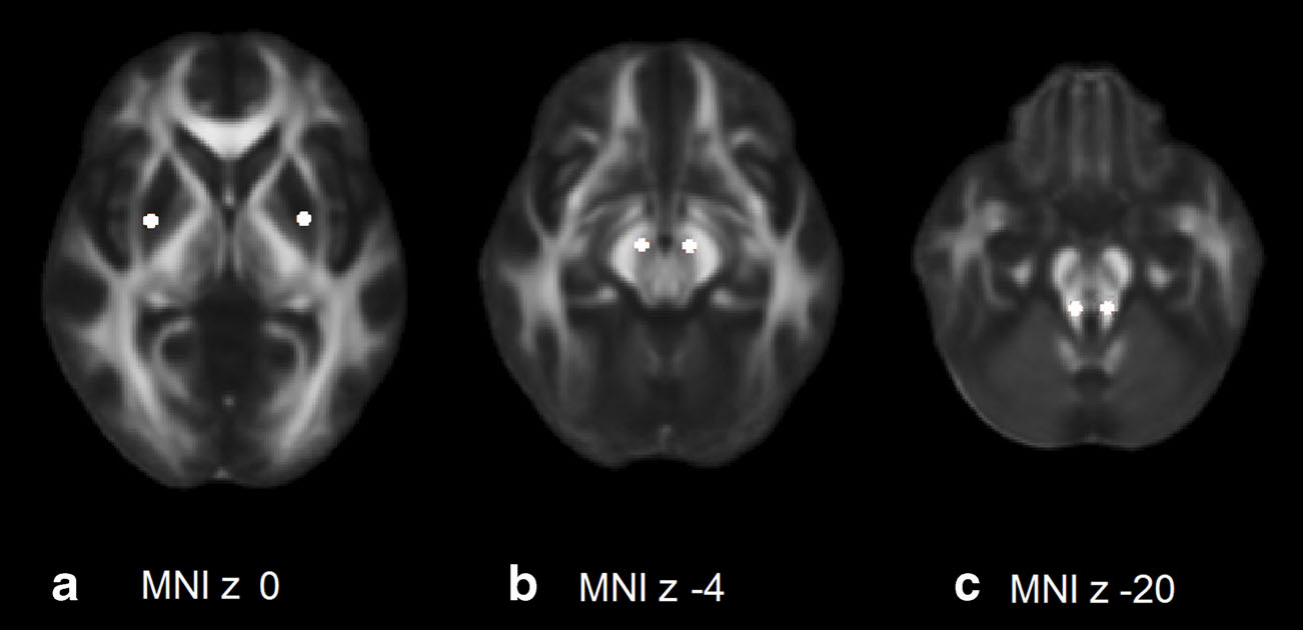
Regions of interest in the bilateral putamen (**a**), midbrain (**b**), and superior cerebellar peduncles (**c**). Mean MD and FA of these ROIs were calculated

Next, a ROI analysis was performed. DTI data were normalized to the MNI space using nonlinear registration with Statistical Parametric Mapping (SPM, Trust Centre of Neuroimaging, London, UK; http://www.fil.ion.ucl.ac.uk/spm/). All images and maps were visually inspected for error or mismatch. Using the FMRIB58_FA standard-space FA template, 16-mm^2^ ROIs were placed in the following gray and white matter structures, which are known to be affected in the different disease entities: bilateral midbrain at the level of the substantia nigra (MNI coordinates 6 and −6, −14, −4), thalamus (10 and −10, −20, 0), putamen (28 and −28, −1,0), caudate nucleus (12 and −12, 18, 0), globus pallidus (16 and −16, 2, −2), superior cerebellar peduncle (SCP) (6 and −6, −36, −20), MCP (20 and −20, −42, −34), dentate nucleus (14 and −14, −56, −30), and the pons (0, −28, −32) (Fig. 1). Matlab (MathWorks, Natick, MA) was used to calculate MD and FA values of these ROIs. ROI placement was visually checked in correlation with conventional brain MRI for each dataset and corrected if necessary.

### Statistical analyses

#### TBSS analysis

A two-sample *t* test using permutation-based statistical interference as a part of FSL toolbox (“randomise”) was performed with 5000 permutation sets, corrected for multiple comparisons across space using the threshold-free cluster enhancement to compare MD and FA of PD with AP and PD with AP subgroups.

#### ROI analysis

Mean MD and FA values of the different ROIs were calculated for each disease group, and one-way ANOVA, corrected for multiple comparisons with a Bonferroni correction, was performed to analyze group differences. A *p* value below 0.05 was considered statistically significant. Threshold values of MD or FA were chosen based on optimal sensitivity and specificity to discriminate AP from PD using receiver operating characteristic (ROC) analyses. The summation of DTI measures above defined thresholds was used to evaluate the diagnostic performance of DTI.

Inter-rater variability of the abnormalities scored on conventional brain MRI was analyzed with the use of Cohen’s kappa coefficient, defined as follows: <0.20, poor agreement; 0.21–0.4, fair agreement; 0.41–0.60, moderate agreement; 0.61–0.80, good agreement; and >0.80, perfect agreement. Diagnostic performance of brain MRI was assessed based on the summation of all abnormal findings. The ROC was used to evaluate the discriminative power of brain MRI alone and combined with selected DTI ROI measures. Statistical analyses were performed with SPSS (IBM SPSS statistics 20).

## Results

### Study group

After clinical follow-up (mean 28.3 ± 8.8 months), probable diagnoses of PD or AP could be made in 49 of the 60 patients: 30 patients were diagnosed with PD and 19 patients with AP. Eleven patients had to be excluded from the analyses for the following reasons: brain MRI with severe artifacts (*n* = 2), uncertain diagnosis (*n* = 6), diagnosis other than PD or AP (*n* = 2), and diagnosis of vascular parkinsonism (*n* = 1). Vascular parkinsonism was excluded because our primary interest was neurodegenerative atypical parkinsonism.

Mean duration of follow-up was longer for PD patients (31.2 ± 6 months) than for AP (23.7 ± 10 months). The group of AP included 12 patients diagnosed MSA-P, 3 patients PSP, 3 patients DLB, and 1 patient CBD. In comparison to PD, the group of AP had a statistically significant longer disease duration (mean 28.4 vs 21.6 months), higher scores of disease severity (H&Y mean score of 2.4 vs 1.7), and severity of motor symptoms (UPDRS-III mean score of 43.5 vs 31.6). Demographic data are summarized in Table 1.

**Table 1:** Patient characteristics

	PD (*n* = 30)	AP (*n* = 19)	*p* value
Age (years)	61.9 (8.1)	65.5 (7.6)	n.s.
Sex (M/F)	17:13	8:11	n.s.
Disease duration (months)	21.6 (11.9)	28.4 (11.1)	*p* = 0.008
UPDRS-III	31.6 (10.2)	43.5 (11.4)	*p* < 0.001
H&Y	1.7 (0.7)	2.4 (0.6)	*p* = 0.001
MMSE	28.4 (1.7)	28.1 (1.6)	n.s.

Mean or number (standard deviation). Student’s *t* test applied to test for group differences

*n.s.* not statistically significant

### DTI analyses

#### Tract-based spatial statistics

**Fig. 2:**
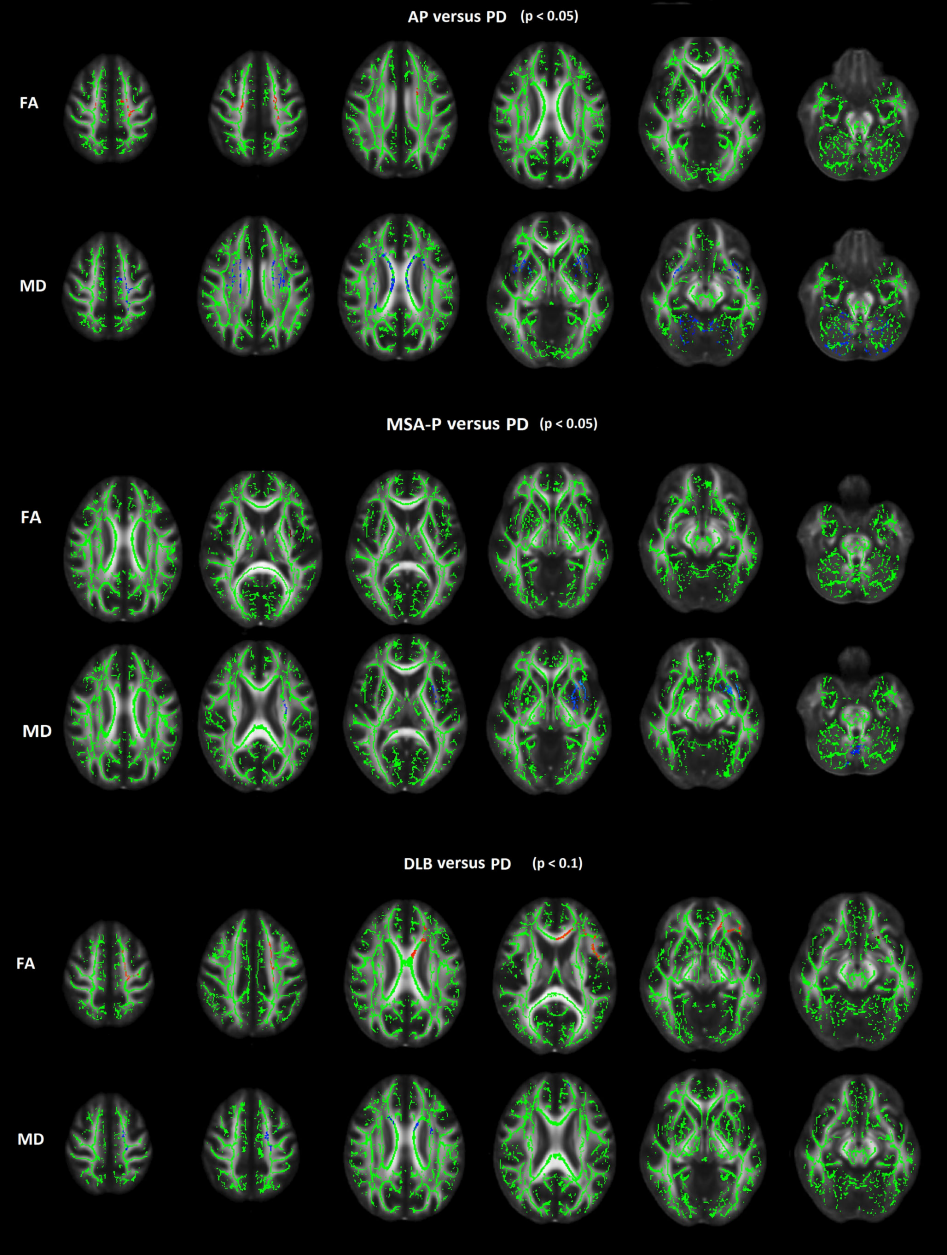
TBSS analyses of atypical parkinsonism with Parkinson’s disease and atypical parkinsonism subgroups with Parkinson’s disease. *Upper row*: TBSS comparison of AP (*n* = 19) with PD (*n* = 29). Brain regions with statistically significant lower FA and higher MD in AP in comparison to PD (*p* < 0.05). *Middle row*: TBSS comparison of MSA-P (*n* = 12) with PD (*n* = 29). MD of the left putamen and external capsule, and superior part of the cerebellar vermis proved to be statistically significant higher for MSA-P in comparison to PD (*p* < 0.05). A part of the left external capsule demonstrated significantly lower FA in MSA-P. *Lower row*: TBSS comparison of DLB (*n* = 3) with PD (*n* = 29). No statistically significant differences were demonstrated, while at a *p* value of <0.1 lower FA and higher MD in the left frontal lobe is seen for DLB in comparison PD. No differences were demonstrated in a TBSS comparison between PSP and PD. *Red*, lower values; *blue*, higher values

Results of TBSS analyses to compare AP (*n* = 19) with PD (*n* = 29) and to compare MSA-P and DLB with PD are shown in Fig. 2. One patient diagnosed with PD had to be excluded because normalization of DTI to the MNI space failed.

A symmetric pattern of bilateral higher MD of the following white matter structures was found for AP in comparison to PD patients (statistically significant, *p* < 0.05): centrum semiovale, body corpus callosum, external capsule, and superior part of the cerebellum. In addition, the subcortical white matter of the left superior frontal gyrus showed significantly higher MD for AP in comparison to PD subjects. Significantly lower FA of the centrum semiovale was found for AP, predominantly on the left side. The other brain structures did not show significant differences in FA between PD and AP.

In a TBSS comparison between MSA-P and PD, MD of the putamen and external capsule on the left side, and the superior part of the cerebellar vermis proved to be statistically significant higher in MSA-P (*p* < 0.05). A part of the left external capsule demonstrated significantly lower FA in MSA-P in comparison to PD. Near statistically significant higher MD of the right putamen and external capsule was observed in MSA-P (*p* < 0.1).

Higher MD and lower FA of the anterior part of the centrum semiovale and genu corpus callosum on the left side was found for DLB in comparison to PD, though this difference was not statically significant (*p* < 0.1). TBSS to compare PSP with PD did not show differences in MD or FA, especially no differences in diffusivity were demonstrated in the superior cerebellar peduncles for PSP.

#### Region of interest analyses

The brain structures which demonstrated significantly higher MD in different forms of AP are summarized in Table 2; mean MD and FA measures of the all brain structures evaluated in our study are provided in the Appendix. MD values of the putamen were significantly higher for MSA-P in comparison to PD, but also higher than the other forms of AP (though not reaching statistical significance). MD values of the left SCP were significantly higher in MSA-P in comparison to PD but comparable to PSP and DLB. In PSP, significantly higher MD values of the midbrain and right SCP were observed in comparison to the other diseases. No diffusional changes were observed for PD or DLB in comparison to the other diseases.

**Table 2:** Statistically significant group differences in ROI measures of MD and FA in Parkinson’s disease and atypical parkinsonism

ROI location	PD (30)	AP (19)			
			MSA-P (12)	PSP (3)	DLB (3)
Putamen right MD*	0.76 (0.14)	0.87 (0.21)	0.92 (0.22)	0.76 (0.16)	0.79 (0.18)
			MSA vs PD *p* = 0.032		
Putamen right FA	0.46 (0.09)	0.44 (0.13)	0.41 (0.15)	0.49 (0.10)	0.50 (0.05)
Putamen left MD*	0.72 (0.11)	0.82 (0.18)	0.87 (0.20)	0.77 (0.04)	0.66 (0.09)
			MSA vs PD *p* = 0.01		
Putamen left FA	0.49 (0.08)	0.46 (0.09)	0.43 (0.10)	0.50 (0.03)	0.54 (0.06)
Midbrain right MD*	0.71 (0.10)	0.75 (0.13)	0.71 (0.13)	0.92 (0.04)	0.73 (0.06)
				PSP vs PD *p* = 0.011	
				PSP vs MSA *p* = 0.022	
Midbrain right FA	0.54 (0.07)	0.56 (0.05)	0.57 (0.06)	0.52 (0.01)	0.56 (0.02)
Midbrain left MD*	0.72 (0.12)	0.86 (0.17)	0.81 (0.11)	1.15 (0.11)	0.77 (0.10)
				PSP vs PD *p* < 0.001	
				PSP vs MSA *p* < 0.001	
				PSP vs DLB *p* = 0.002	
Midbrain left FA	0.56 (0.08)	0.52 (0.8)	0.55 (0.08)	0.44 (0.01)	0.50 (0.08)
SCP right MD*	0.91 (0.15)	1.04 (0.23)	0.96 (0.23)	1.32 (0.01)	1.04 (0.11)
				PSP vs PD *p* = 0.001	
				PSP vs MSA *p* = 0.011	
SCP right FA	0.70 (0.07)	0.65 (0.10)	0.67 (0.12)	0.58 (0.02)	0.65 (0.09)
SCP left MD*	0.86 (0.10)	0.99 (0.19)	1.01 (0.21)	0.97 (0.07)	0.92 (0.18)
			MSA vs PD *p* = 0.018		
SCP left FA	0.69 (0.08)	0.66 (0.11)	0.65 (0.12)	0.66 (0.06)	0.71 (0.07)

Mean (standard deviation) MD (×10^−3^ mm^2^/s) and FA values of ROI in different brain structures for PD, MSA-P, PSP, and DLB. One-way ANOVA (corrected for multiple comparisons with a Bonferroni correction) to analyze group differences between (*p value below 0.05 considered statistically significant). Structures with statistically significant differences are shown in this table, see Appendix Table 4 for all structures which have been evaluated

*PD* Parkinson’s disease, *AP* neurodegenerative atypical parkinsonism, *MSA-P* multiple system atrophy–parkinsonian form, *PSP* progressive supranuclear palsy, *DLB* dementia with Lewy bodies, *SCP* superior cerebellar peduncle, *MD* mean diffusivity, *FA* fractional anisotropy

**Fig. 3:**
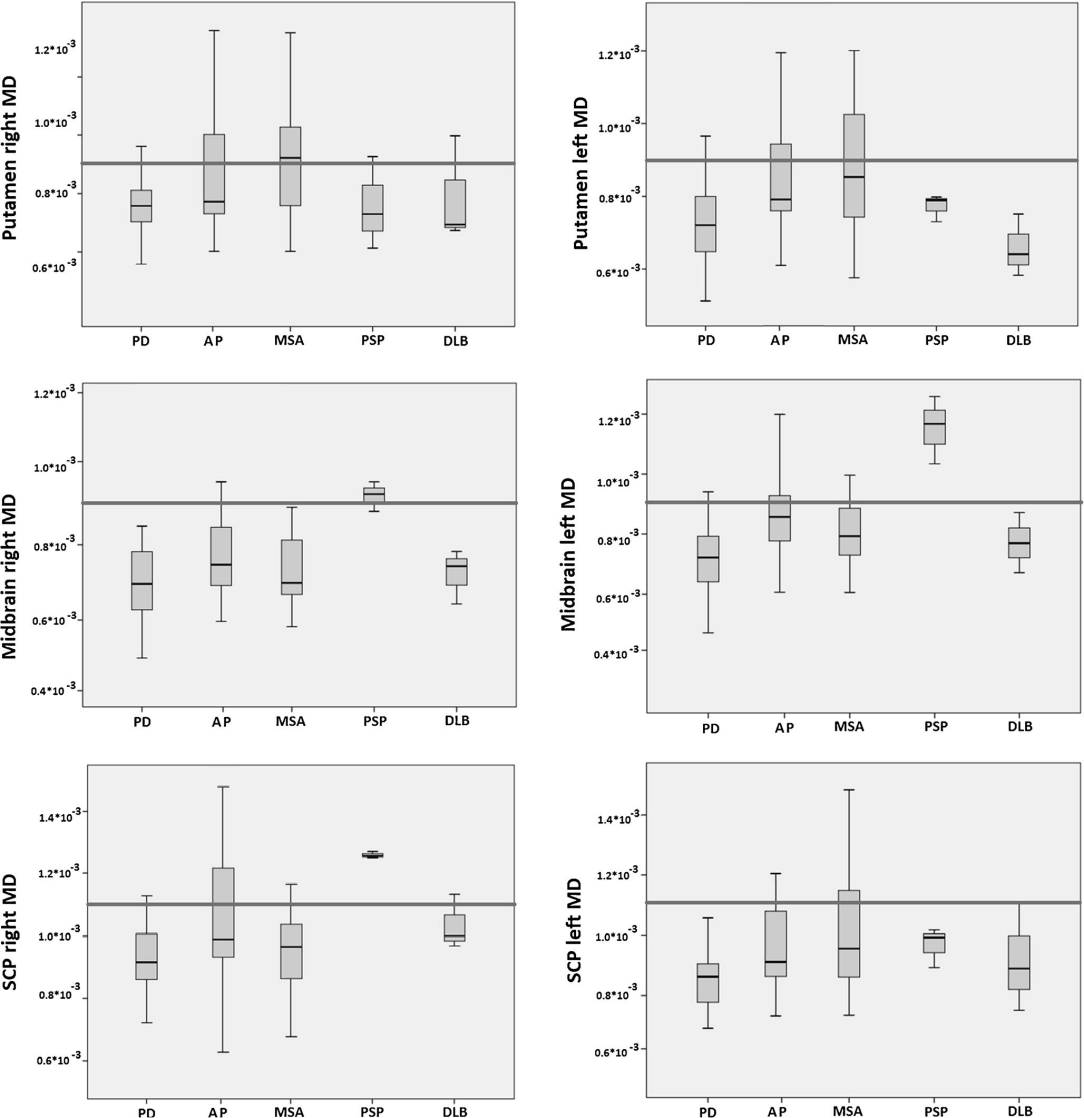
Boxplots of MD values (mm^2^/s) of the putamen, midbrain, and SCP. *Horizontal lines* indicate the defined threshold MD values to discriminate AP from PD (putamen and midbrain MD values of 0.9 × 10 and 1.1 × 10^−3^ mm^2^/s for the SCP). *PD* Parkinson’s disease, *AP* neurodegenerative atypical parkinsonism, *MSA-P* multiple system atrophy–parkinsonian form, *PSP* progressive supranuclear palsy, *DLB* dementia with Lewy bodies, *SCP* superior cerebellar peduncle

Figure 3 shows boxplots of the distribution of MD values for the different diseases. Based on additional ROC analyses to determine optimal sensitivity and specificity, threshold MD values were determined to discriminate AP from PD: MD value of 0.9 × 10^−3^ mm^2^/s for the putamen and midbrain, and 1.1 × 10^−3^ mm^2^/s for the SCP. Subjects with MD values above one of these thresholds consisted mainly of patients diagnosed with AP. The MD cutoff thresholds for these structures were applied to our cohort and the summation of results was used for the evaluation of the diagnostic accuracy of DTI to identify AP.

### Diagnostic accuracy of brain MRI and DTI

Abnormalities scored on conventional brain MRI are summarized in Table 3. As described earlier, the putaminal rim sign noted on 3 T brain MRI is a normal finding and not indicative of AP [27]. In our study, we confirm that this finding has no diagnostic value in the identification of MSA-P (sensitivity 33 %, specificity 51 %) or AP (sensitivity 37 % and specificity 50 %). This was the reason that we did not include the putaminal rim sign in our analysis to evaluate the performance of conventional brain MRI.

**Table 3:** Frequency of abnormalities on conventional brain MRI

Abnormality	PD (30), *N* (%)	AP (19), *N* (%)					Inter-rater variability (kappa)
			MSA-P (12), *N* (%)	PSP (3), *N* (%)	DLB (3), *N* (%)	CBD (1), *N* (%)	
Putaminal atrophy	4 (13)	3 (16)	3 (25)	0 (0)	0 (0)	0 (0)	0.92
Putaminal T2 hypo-intensity	2 (7)	9 (47)	7 (58)	1 (33)	1 (33)	0 (0)	0.94
Putaminal rim	15 (30)	7 (37)	4 (33)	1 (33)	1 (33)	1(100)	0.00
Pons atrophy	1 (3)	2 (11)	2 (17)	0 (0)	0 (0)	0 (0)	0.48
Hot cross bun sign	0 (0)	0 (0)	0 (0)	0 (0)	0 (0)	0 (0)	1.00
Cerebellar atrophy	4 (13)	6 (32)	3 (25)	0 (0)	3 (100)	0 (0)	0.46
MCP T2 hyper-intensity	0 (0)	3 (16)	3 (25)	0 (0)	0 (0)	0 (0)	0.65
Midbrain Atrophy	0 (0)	3 (16)	1 (5)	2 (67)	0 (0)	0 (0)	0.73
Hummingbird sign	0 (0)	4 (21)	1 (5)	3 (100)	0 (0)	0 (0)	1.00
Midbrain <14 mm	0 (0)	3 (16)	1 (5)	2 (67)	0 (0)	0 (0)	0.65
Cortical atrophy	10 (33)	9 (47)	5 (26)	1 (33)	3 (100)	0 (0)	0.62
Dilatation third ventricle	5 (17)	12 (63)	7 (58)	3 (100)	2 (67)	0 (0)	0.67
Dilatation lateral ventricles	4 (13)	8 (42)	4 (33)	2 (67)	2 (67)	0 (0)	0.94
Infarction	2 (7)	1 (5)	0 (0)	0 (0)	1 (33)	0 (0)	0.64
Confluent white matter changes	4 (13)	1 (5)	1 (5)	0 (0)	0 (0)	0 (0)	0.62

*PD* Parkinson’s disease, *AP* neurodegenerative atypical parkinsonism, *MSA-P* multiple system atrophy–parkinsonian form, *PSP* progressive supranuclear palsy, *DLB* dementia with Lewy bodies, *CBD* corticobasal degeneration, *MCP* middle cerebellar peduncle

Atrophy and T2 hypo-intensity of the putamen, the hummingbird sign, and lateral ventricle dilatation proved to have perfect inter-rater agreement (kappa >0.92). Inter-rater agreement was good for T2 hyper-intensity changes of the middle cerebellar peduncle, cortical atrophy, and third ventricle dilatation (kappa 0.62–0.73) while inter-rater agreement was moderate for atrophy of the pons and cerebellum (kappa 0.46–0.48).

**Fig. 4:**
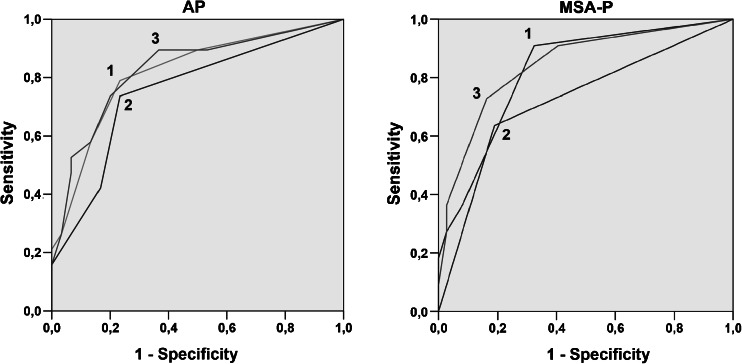
ROC curves of brain MRI and DTI to identify neurodegenerative atypical parkinsonism as a group (*left*) and MSA-P (*right*). Conventional brain MRI (*1*), DTI (*2*), and MRI with DTI combined (*3*). Quantitative DTI measures of DTI did not increase the diagnostic accuracy of brain MRI for the diagnosis of AP as a group: AUC 0.83 (95 % CI 0.70–0.95). The AUC for the MRI diagnosis of MSA-P was slightly increased by MD of the putamen: AUC 0.82 (95 % CI 0.69–0.96) was increased to 0.85 (95 % CI 0.71–0.98). *AUC* area under the curve, *CI* confidence interval, *MSA-P* multiple system atrophy–parkinsonian form

The diagnostic accuracy of brain MRI to identify AP resulted in an AUC of 0.82 (95 % CI 0.69–0.94) and for DTI in an AUC of 0.75 (95 % CI 0.61–0.90), as demonstrated in Fig. 4. The combination of brain MRI and DTI resulted in an AUC of 0.83 (95 % CI 0.70–0.95). DTI therefore did not significantly improve the diagnostic accuracy of brain MRI to differentiate the group of AP from PD. Abnormalities on brain MRI considered to be specific for MSA-P resulted in an AUC of 0.82 (95 % CI 0.69–0.96) to identify MSA-P. The AUC was slightly increased to 0.85 (95 % CI 0.71–0.98) when combined with MD of the putamen.

## Discussion

We evaluated the diagnostic accuracy of 3 T brain MRI and DTI to differentiate AP from PD in early stage parkinsonism. Unlike previous studies, we evaluated brain MRI performed at baseline in a cohort of patients with initial uncertain clinical diagnosis. TBSS demonstrated higher MD of the centrum semiovale, external capsule, putamen, and superior cerebellum in AP in comparison to PD. FA of the centrum semiovale was significantly lower in AP. This pattern of differences in diffusivity probably represents the summation of microstructural changes of different disease entities in the AP group. In MSA-P, MD of the left putamen, left external capsule, and superior part of the cerebellar vermis proved to be statistically significant higher in comparison to PD, with lower FA in a part of the left external capsule.

Results of the TBSS and the ROI methods showed some discrepancies, such as significantly higher MD values of the SCP and midbrain in PSP demonstrated with the ROI method but not confirmed by TBSS. For other brain structures, results were in accordance such as higher MD of the putamen in MSA-P. Results of these two methods were therefore considered complementary rather than contradictory.

The diagnostic accuracy of brain MRI to identify AP as a group was not improved when combined with the ROI measurements of MD in the putamen, midbrain, and SCP, although the AUC was slightly increased when evaluating the diagnostic accuracy to identify the subgroup of MSA-P. Disease specific diagnostic measures of DTI probably give a better estimation of the diagnostic accuracy rather grouping all the different forms of AP together. This is illustrated by our ROI analyses where differences in MD of the putamen or midbrain can be identified in MSA-P or PSP, while the averaged MD for AP as a group does not statistically differ from PD.

We found significantly increased putaminal MD values for MSA-P in comparison to PD. Diffusional changes of the putamen in MSA correspond to known underlying neuropathologic changes in the nigrostriatal system, with more severe involvement of the posterior part of the putamen compared to its anterior part in MSA-P, as has been reported in previous studies [28–33]. The chosen putaminal MD cutoff threshold of 0.9 × 10^−3^ mm^2^/s is in accordance with other studies [32, 33].

Increased MD values in the midbrain and SCP in PSP as compared to both MSA and PD, as we found, have also been reported previously [34–38]. Histopathologic changes in PSP include damage in the cerebellar dentate nucleus and its projection fibers in the SCP [39]. The clinical significance of damage to the SCP in PSP was found uncertain, and degeneration of the SCP appears unrelated to disease duration or typical clinical findings such as gaze palsy and postural instability [40]. It has been reported that midbrain diffusional changes in PSP seem to correlate with disease progression [41], while in our study, this was observed already in early disease stages.

We did not find altered diffusional measures in subcortical gray matter structures in DLB. Possibly, white matter structures in the left frontal lobe and corpus callosum could be affected in DLB, though the difference in MD and FA values between PD and DLB found by our TBSS analyses were not statistically significant. There is a debate whether DLB and dementia in PD (PDD) are the same disease entities [42]. Subtle cognitive deficits indicating frontal lobe dysfunction are common in early stage PD, while PDD frequently occurs in late stages. Contradictory study results have been published for DTI studies comparing DLB with PDD [43–46], though these discrepancies could be attributed to differences in scanning protocols and MRI field strengths. It remains to be determined whether DTI could provide diagnostic markers to identify DLB.

As many studies on DTI in parkinsonism focused on group analyses and evaluating patients in advanced disease stages [10], the challenge now lies in clinical application of quantitative DTI in the diagnostic workup of an individual patient presenting with parkinsonism. A major advantage of TBSS over the ROI method is that it enables a hypothesis-free analysis of whole brain DTI. Although effects of misalignment due to registration and smoothing are limited, TBSS is criticized for problems with the required registration process and less reliable estimation of the diffusivity at multiple fiber orientation, such as crossing fibers. Drawbacks of TBSS are that it is only suited for the evaluation of major white matter tracts and not for gray matter structures, and diagnostic criteria for clinical use are not readily provided which hinders the evaluation of an individual patient. Quantitative measures of diffusivity of an individual patient can easily be performed with the ROI method, but for use in clinical practice validated diagnostic criteria should be applied. Error correction and standardized ROI placement is warranted for reliable and reproducible quantitative DTI analysis and this should not be cumbersome. Only few studies evaluated DTI in relation to conventional brain MRI or other advanced MRI techniques. Focke et al. studied DTI in relation to R2* and based on their results R2* seems to be of value for the diagnosis of MSA and DTI for the diagnosis of PSP [34].

Although diffusional changes are considered to represent a quantitative measure of neurodegenerative changes in the brain, changes are notoriously difficult to interpret due to an insufficient understanding of the structural underpinnings of these changes. For example, it can be debated whether diffusional changes are the representation of the primary pathologic process, the result of a secondary consequence or age-related diffusional changes [47]. Diffusion MRI has been used to get a better understanding of specific nonmotor signs of PD such as hyposmia and depression [48–51], but those probably represent a secondary consequence. Previous study results are conflicting whether or not levadopa treatment would be of influence on changes in diffusivity of different brain structures. One study reported significant differences in putaminal ADC values between PD patients on levodopa treatment and matched untreated patients [52], and they suggested that these differences could be attributed to the use of levodopa. However, other studies found no effect of levodopa treatment on FA or ADC values [53, 54].

The complexity of interpreting diffusional changes is illustrated by two recently published systematic reviews, which differed in their conclusion whether DTI of the substantia nigra can be used as a diagnostic marker for PD [8, 9]. Cochrane et al. found highly significant PD induced FA reduction in the substantia nigra [8]. On the contrary, Schwarz et al. concluded that there is insufficient evidence for nigral DTI measures to serve as a useful diagnostic marker of PD at this point in time [9]. Differences in studies included in these two meta-analyses as well as a variation of extracted values from included studies could explain their contradicting conclusions. In our study, FA and MD values of the midbrain in PD were comparable to MSA-P and DLB, and in our TBSS analyses, no PD-specific DTI changes could be demonstrated. It remains a debate whether or not DTI would provide a new diagnostic measure for clinical use to identify PD in the early disease stages.

There are some limitations to our study:

First, our study population was relatively small, especially in relation to the number of different MRI parameters studied. It consisted of patients presenting with predominantly hypo-kinetic symptoms and uncertain clinical diagnosis, probably explaining the majority of AP patients diagnosed with MSA-P and the low prevalence of other forms of neurodegenerative AP. It has been demonstrated that the added value of brain MRI in the diagnostic workup of parkinsonism is highest for those patients where the baseline certainty about the diagnosis is lowest [6]. Based on initial clinical evaluation, patients with probable diagnoses of PSP, DLB, CBS, and vascular parkinsonism were excluded from the study. The classic phenotype of PSP, now called Richardson’s syndrome, is characterized by early onset postural instability and falls, supranuclear gaze palsy, and cognitive dysfunction [55]. It is the parkinsonism form of PSP, dominated by asymmetric onset, tremor, and moderate initial therapeutic response to levodopa, which renders the differentiation with PD difficult [56]. The same accounts for corticobasal syndrome, which is suspected when cortical dysfunction is prominent (e.g., alien limb phenomenon, cognitive decline, or behavioral abnormalities), while the differentiation with PD can be difficult in case of presence of asymmetric parkinsonism and rigidity [57, 58]. Clinical presentation of vascular parkinsonism usually includes postural instability and falls, rather than upper limb rest tremor or bradykinesia [59]. As a consequence of our inclusion, our study is underpowered to draw definite conclusions whether DTI is of added value for the diagnosis of separate AP subgroups. On the other side, prevalence of less frequent diseases does reflect clinical practice and illustrates the challenges for ancillary investigations to identify more rare causes of parkinsonism. The new element of our study is that we evaluated whether DTI improves the diagnostic accuracy of brain MRI to identify AP as a group in case of uncertainty about the clinical diagnosis, where it is of the most clinical relevance. Our patient cohort did not include patients diagnosed with MSA-C (clinical presentation distinct from PD and MSA-P with predominant cerebellar symptoms), which could explain that no significant diffusion differences of the MCP and pons were found in MSA-P, although diffusional changes in these structures have also been reported in MSA-P [32, 33, 60]. Although vascular parkinsonism was beyond the scope of our study, DTI could be of value for the diagnosis of vascular parkinsonism [61, 62]. Further studies are warranted and should include a larger sample size to evaluate the additional value of DTI for improved differentiation between the various atypical parkinsonism subtypes in the early disease stages.

Second, we did not have post mortem confirmation to reach the gold standard diagnosis and cannot fully exclude misdiagnosis. Clinical follow-up enabled us to improve certainty of the diagnosis. It has been shown that in the hands of an experienced movement disorder specialist, clinical follow-up for at least 2 years enables accurate diagnosis by evaluating the rate of disease progression, treatment response and development of any red flags [5].

Third, interpretation of the reported quantitative MD and FA values should be done with caution because comparisons of diffusional values in different studies is difficult as scanning protocols and post processing of diffusional data lack standardization. It is known that MD and FA values vary with the MRI field strength used [63]. As DTI is sensitive to susceptibility changes, FA and MD values are probably influenced by brain iron accumulation and calcification [9]. Future studies need to elucidate to what extent quantitative diffusion analysis should be corrected for tissue susceptibility changes, taking the MRI field strength into account. Furthermore, fractional anisotropy is derived from the first, second, and third eigenvectors (and subsequent eigenvalues), which could provide additional measures of microstructural integrity of brain tissue. Parallel imaging and other accelerating techniques enable acquisition of high resolution DTI [64], which could be superior for the detection of more subtle neurodegenerative changes at acceptable scanning times to enable clinical application but this needs to be determined in future prospective clinical cohort studies. In a case-control study evaluating subjects with PD, it has been reported that diffusional kurtosis imaging, which enables the quantification of non-Gaussian diffusion, is a more sensitive technique than conventional DTI for assessing tissue microstructure, even in the presence of crossing fibers [65].

Finally, although the diagnostic accuracy of brain MRI in our study was not improved by DTI using the current analyses approach, we cannot exclude that it will prove to be of added value for the diagnostic work-up of parkinsonism while using a different methods of analysis. A previous study using a slightly different ROI approach with multiple DTI measures in the basal ganglia and cerebellum reported high accuracy in classifying patients with PD, MSA-P, PSP and control subjects [11]. The ROI method is restricted because only a few ROIs are chosen based on a priori hypothesis and diffusional changes outside the ROI are not analyzed. Furthermore, it bears the pitfall of partial volume averaging of MD and FA measurements. Although whole brain analyses were performed with TBSS, this method is less suitable for evaluating gray matter structures as has been discussed earlier.

Machine-learning algorithms have been developed for advanced MR imaging techniques, and initial results of applying this technique to analyze DTI in a cohort of patients with parkinsonism are promising [66]. These machine-learning techniques rely on algorithms analyzing imaging data without a priori hypotheses, based on which classifiers can be constructed for pattern recognition at the individual level [67, 68]. Compared with a single imaging technique, the advantage of using multiple techniques is to extract more features in order to more accurately profile specific neurodegenerative pathology [68].

## Conclusion

In early stage parkinsonism, distinct brain regions showed higher mean diffusivity and lower fractional anisotropy values in the atypical parkinsonism group as compared to Parkinson’s disease. Using a ROI approach, increased MD measures of the putamen, midbrain, and superior cerebellar peduncles seem appropriate for differentiating AP from PD. The diagnostic accuracy of brain MRI in the identification of atypical parkinsonism as a group was not improved by the current analysis approach to DTI, though it could be of added value to identify atypical parkinsonism subgroups.

## Appendix

**Table 4:** ROI measures of MD and FA in Parkinson’s disease and atypical parkinsonism

ROI location	PD (30)	AP (19)			
			MSA-P (12)	PSP (3)	DLB (3)
Caudate nucleus right MD	0.77 (0.16)	0.73 (0.18)	0.66 (0.17)	0.94 (0.06)	0.82 (0.11)
Caudate nucleus right FA	0.43 (0.07)	0.47 (0.09)	0.50 (0.11)	0.42 (0.03)	0.44 (0.03)
Caudate nucleus left MD	0.93 (0.21)	0.95 (0.25)	0.86 (0.22)	1.24 (0.18)	1.10 (0.25)
Caudate nucleus left FA	0.37 (0.07)	0.39 (0.07)	0.43 (0.06)	0.30 (0.05)	0.37 (0.09)
Putamen right MD*	0.76 (0.14)	0.87 (0.21)	0.92 (0.22)	0.76 (0.16)	0.79 (0.18)
			MSA vs PD *p* = 0.032		
Putamen right FA	0.46 (0.09)	0.44 (0.13)	0.41 (0.15)	0.49 (0.10)	0.50 (0.05)
Putamen left MD*	0.72 (0.11)	0.82 (0.18)	0.87 (0.20)	0.77 (0.04)	0.66 (0.09)
			MSA vs PD *p* = 0.01		
Putamen left FA	0.49 (0.08)	0.46 (0.09)	0.43 (0.10)	0.50 (0.03)	0.54 (0.06)
GP right MD	0.77 (0.23)	0.79 (0.23)	0.75 (0.22)	0.84 (0.07)	0.93 (0.36)
GP right FA	0.54 (0.11)	0.54 (0.11)	0.56 (0.09)	0.56 (0.02)	0.53 (0.16)
GP left MD	0.76 (0.23)	0.55 (0.09)	0.83 (0.25)	0.65 (0.05)	0.86 (0.32)
GP left FA	0.56 (0.11)	0.54 (0.11)	0.53 (0.11)	0.61 (0.05)	0.52 (0.14)
Thalamus right MD	0.71 (0.06)	0.76 (0.12)	0.76 (0.14)	0.73 (0.08)	0.77 (0.11)
Thalamus right FA	0.53 (0.06)	0.53 (0.08)	0.53 (0.10)	0.57 (0.05)	0.51 (0.06)
Thalamus left MD	0.70 (0.08)	0.71 (0.09)	0.71 (0.10)	0.72 (0.10)	0.70 (0.05)
Thalamus left FA	0.50 (0.06)	0.50 (0.08)	0.49 (0.09)	0.54 (0.03)	0.53 (0.05)
Midbrain right MD*	0.71 (0.10)	0.75 (0.13)	0.71 (0.13)	0.92 (0.04)	0.73 (0.06)
				PSP vs PD *p* = 0.011	
				PSP vs MSA *p* = 0.022	
Midbrain right FA	0.54 (0.07)	0.56 (0.05)	0.57 (0.06)	0.52 (0.01)	0.56 (0.02)
Midbrain left MD*	0.72 (0.12)	0.86 (0.17)	0.81 (0.11)	1.15 (0.11)	0.77 (0.10)
				PSP vs PD *p* < 0.001	
				PSP vs MSA *p* < 0.001	
				PSP vs DLB *p* = 0.002	
Midbrain left FA	0.56 (0.08)	0.52 (0.8)	0.55 (0.08)	0.44 (0.01)	0.50 (0.08)
Pons MD	0.75 (0.12)	0.72 (0.13)	0.69 (0.15)	0.73 (0.05)	0.79 (0.12)
Pons FA	0.52 (0.07)	0.54 (0.08)	0.55 (0.09)	0.55 (0.06)	0.51 (0.11)
SCP right MD*	0.91 (0.15)	1.04 (0.23)	0.96 (0.23)	1.32 (0.01)	1.04 (0.11)
				PSP vs PD *p* = 0.001	
				PSP vs MSA *p* = 0.011	
SCP right FA	0.70 (0.07)	0.65 (0.10)	0.67 (0.12)	0.58 (0.02)	0.65 (0.09)
SCP left MD*	0.86 (0.10)	0.99 (0.19)	1.01 (0.21)	0.97 (0.07)	0.92 (0.18)
			MSA vs PD *p* = 0.018		
SCP left FA	0.69 (0.08)	0.66 (0.11)	0.65 (0.12)	0.66 (0.06)	0.71 (0.07)
MCP right MD	0.64 (0.07)	0.66 (0.08)	0.64 (0.10)	0.67 (0.03)	0.68 (0.06)
MCP right FA	0.72 (0.06)	0.68 (0.08)	0.70 (0.09)	0.69 (0.04)	0.63 (0.06)
MCP left MD	0.65 (0.05)	0.66 (0.09)	0.68 (0.09)	0.66 (0.08)	0.61 (0.11)
MCP left FA	0.71 (0.06)	0.70 (0.07)	0.69 (0.07)	0.76 (0.05)	0.70 (0.09)
Dentate nucleus right MD	0.73 (0.10)	0.76 (0.13)	0.72 (0.10)	0.83 (0.10)	0.88 (0.21)
Dentate nucleus right FA	0.54 (0.07)	0.51 (0.07)	0.53 (0.07)	0.47 (0.06)	0.48 (0.08)
Dentate nucleus left MD	0.71 (0.09)	0.70 (0.14)	0.68 (0.18)	0.72 (0.01)	0.74 (0.04)
Dentate nucleus left FA	0.52 (0.06)	0.52 (0.06)	0.54 (0.06)	0.50 (0.02)	0.49 (0.05)

Mean (standard deviation) MD (×10^−3^ mm^2^/s) and FA values of ROI in different brain structures for PD, MSA-P, PSP, and DLB. One-way ANOVA (corrected for multiple comparisons with a Bonferroni correction) to analyze group differences between (**p* value below 0.05 considered statistically significant)

*PD* Parkinson’s disease, *AP* neurodegenerative atypical parkinsonism, *MSA-P* multiple system atrophy–parkinsonian form, *PSP* progressive supranuclear palsy, *DLB* dementia with Lewy bodies, *GP* globus pallidus, *MCP* middle cerebellar peduncle, *SCP* superior cerebellar peduncle, *MD* mean diffusivity, *FA* fractional anisotropy

## Abbreviations

PD: Parkinson’s disease
AP: Neurodegenerative atypical parkinsonism
MSA-P: Multiple system atrophy–parkinsonian form
MSA-C: Multiple system atrophy–cerebellar form
PSP: Progressive supranuclear palsy
DLB: Dementia with Lewy bodies
CBS: Corticobasal syndrome
MCP: Middle cerebellar peduncle
SCP: Superior cerebellar peduncle
ROC: Receiver operating characteristic
AUC: Area under the curve
CI: Confidence interval
DTI: Diffusion tensor imaging
FA: Fractional anisotropy
MD: Mean diffusivity
